# Nativity differences in socioeconomic barriers and healthcare delays among cancer survivors in the All of Us cohort

**DOI:** 10.1007/s10552-023-01782-z

**Published:** 2023-09-07

**Authors:** Angel Arizpe, Stephanie Navarro, Carol Y. Ochoa-Dominguez, Claudia Rodriguez, Sue E. Kim, Albert J. Farias

**Affiliations:** 1 Keck School of Medicine of the University of Southern California, 2001 N. Soto St., Suite 318B, Los Angeles, CA 90032, USA; 2 University of California, San Diego, San Diego, CA, USA; 3 University of Southern California, Los Angeles, CA, USA

**Keywords:** SES barriers, Healthcare delays, Nativity, All of Us, Cancer survivors, Health literacy

## Abstract

**Purpose:**

We aimed to assess whether nativity differences in socioeconomic (SES) barriers and health literacy were associated with healthcare delays among US cancer survivors.

**Methods:**

“All of Us” survey data were analyzed among adult participants ever diagnosed with cancer. A binary measure of healthcare delay (1+ delays versus no delays) was created. Health literacy was assessed using the Brief Health Literacy Screen. A composite measure of SES barriers (education, employment, housing, income, and insurance statuses) was created as 0, 1, 2, or 3+. Multivariable logistic regression model tested the associations of (1) SES barriers and health literacy with healthcare delays, and (2) whether nativity modified this relationship.

**Results:**

Median participant age was 64 years (*n* = 10,020), with 8% foreign-born and 18% ethnic minorities. Compared to survivors with no SES barriers, those with 3+ had higher likelihood of experiencing healthcare delays (OR 2.18, 95% CI 1.84, 2.58). For every additional barrier, the odds of healthcare delays were greater among foreign-born (1.72, 1.43, 2.08) than US-born (1.27, 1.21, 1.34). For every 1-unit increase in health literacy among US-born, the odds of healthcare delay decreased by 9% (0.91, 0.89, 0.94).

**Conclusion:**

We found that SES barriers to healthcare delays have a greater impact among foreign-born than US-born cancer survivors. Higher health literacy may mitigate healthcare delays among US cancer survivors. Healthcare providers, systems and policymakers should assess and address social determinants of health and promote health literacy as a way to minimize healthcare delays among both foreign- and US-born cancer survivors.

## Background

According to the National Cancer Institute (NCI), an individual is considered a cancer survivor from diagnosis to the end of life [1]. In the United States (US), it is estimated that by 2026, the number of cancer survivors will surpass 20 million, which can be attributed to the ongoing innovation of treatment and early disease detection [2, 3]. While this increase in survival is optimistic, this population requires additional healthcare services to prevent or manage chronic health conditions, sequelae of cancer treatment, and monitoring for cancer reoccurrence [3]. Moreover, given that at least 50% of cancer survivors will experience physical and mental health consequences due to their disease or treatment [4], is important to mitigate healthcare delays in this population.

Overall, healthcare delays (due to the domains of accessibility, financial burden, and social support) can significantly impact cancer survivors, most notably individuals who are ethnic minorities, lower SES, or uninsured [5]. Cancer survivors who experience healthcare delays can significantly suffer the consequences as their care is typically time sensitive [6]. Because timely cancer care is associated with a favorable prognosis, barriers or delays to treatment can result in a more advanced stage of cancer at the time of eventual care, thus, resulting in poorer outcomes [7]. However, physical health is not the only aspect affected; mental health may also suffer at the hands of delayed care [8]. For instance, COVID-19 led to numerous appointment cancelations for cancer survivor patients, including self-cancelations that were caused by depression and anxiety symptoms surrounding the pandemic and safe access to care [9]. These missed appointments, in turn, exacerbated patients’ fears of cancer recurrence as their follow-up care were halted (e.g., laboratory testing, imaging, and appointments), impacting their overall well-being and physical and mental health [10, 11].

One factor associated with increases in healthcare delays among cancer survivors is lower SES status, which is associated with all-cause mortality risk, poorer mental (e.g., depression), and physical health outcomes [12, 13]. Similarly, SES has been linked to a range of cancer outcomes and higher SES (suggesting more financial resources and the ability to afford medical care) is associated with decreased in the length of healthcare delays [14]. Because of the long-term and specialized care needed for cancer survivors, they are at risk of experiencing higher financial burden, which in turn, impacts receipt of survivorship care and increases the risk of mortality, and worsens quality of life [15].

A second factor associated with healthcare delays among cancer survivors is a lack of health literacy, which presents a barrier in properly understanding, communicating, and obtaining information required to navigate the complexity of the healthcare systems efficiently to obtain the care needed and to make educated health decisions [16, 17]. Among the general population, low health literacy has been positively associated with various care delays, including seeking treatment, forgoing care, and struggling with accessibility to needed care and providers [18]. As cancer survivors have complex healthcare needs, mastering the skills required for their continual care is important.

Being foreign-born (immigrant) brings an additional barrier to accessing healthcare and consequently promotes healthcare delays [19]. These barriers among foreign-born may be partially attributed to a higher likelihood of lack of insurance, healthcare cultural perception, and English skills proficiency [20]. Evidence suggests that cancer survivors who are immigrants have a lower quality of life and higher depression symptomology compared to native-born [21]. Moreover, foreign-born individuals may experience unique barriers (e.g., language, discrimination, laws and regulations to qualify for services) that impact the quality of care they can receive. For instance, language barriers and insurance difficulties caused by laws and policies may make it much more difficult for an immigrant patient than a US-born patient to receive adequate needed care [22].

**Fig. 1:**
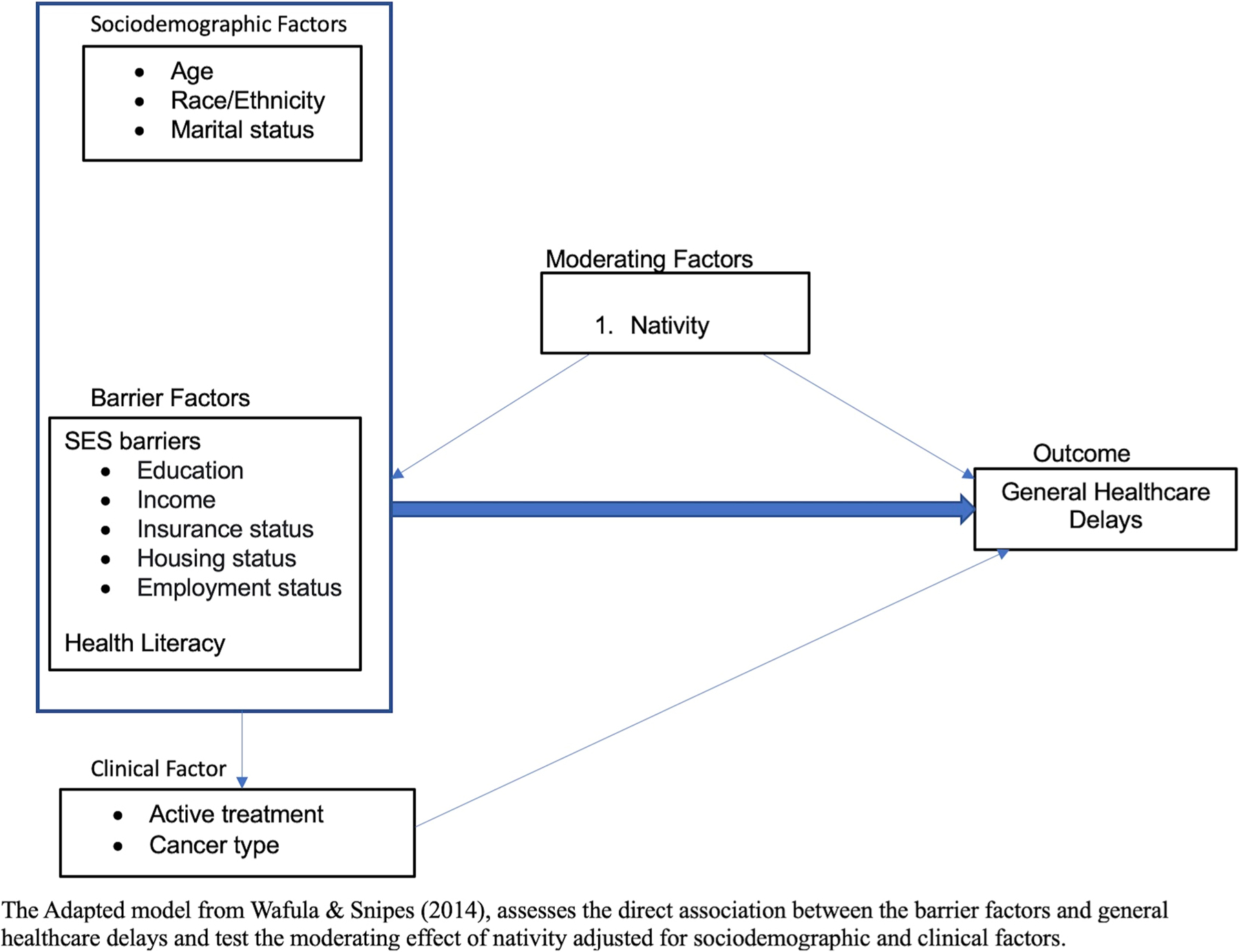
Theoretical framework of the impact nativity has on healthcare delays

While it is well established that SES barriers and a lack of health literacy are associated with healthcare delays, there is limited and inconsistent knowledge of how these associations differ by nativity. To guide the selection of our variables for our model, we used the theoretical framework from Wafula and Snipes for barriers to healthcare among Black immigrants in the US [23]. Additionally, we adapted their framework to focus on assessing the association between the barrier factors (i.e., SES barriers, health literacy) and general healthcare delays among cancer survivors and tested the moderating effects of nativity between SES barriers and health literacy with general healthcare delays (Fig. 1). Thus, this study aims to contribute to the current literature by examining whether nativity status modifies the relationship between a combination of SES and health literacy barriers, and healthcare delays in a large national cohort.

## Methods

### Data collection and sample

Cross-sectional data for this study were obtained from the “All of Us” research program collected by online survey between May 2018 and April 2021. Briefly, this program is open to individuals who are 18 and over and are living in the US. Participants signed a consent form following the Declaration of Helsinki for data collection. The participants’ data used are de-identified and available to approved researchers. The All of Us program was approved by the National Institutes of Health (NIH) Institutional Review Board (IRB).

In this study, cancer survivors were defined as those participants who indicated that they had ever been diagnosed with cancer. Inclusion criteria for our cohort included participants who were ever told by their healthcare provider that had/have cancer. Skin cancer is one of the most prevalent cancers in the US with most cases being reasonably benign basal cells, not often tracked on most cancer registries, and having over 90% 5-year survival rate [24]. In addition, previous studies have excluded these cancer survivors as their follow-up care is often reasonably minor [25]. Thus, we excluded those with skin cancer and participants with missing data on the healthcare delay survey questions.

## Measures

### Demographics

Personal level characteristics accounted for in this study were age (at survey completion), sex (male vs female), race (Asian, Black, Hispanic, White, multiracial/biracial, other [includes those who selected: none of this, another population, and prefer not to answer]), marital status (married [includes those who selected: married and living with a partner] vs single [includes those who selected: Single, divorced, widowed, and separated]), nativity (US- vs foreign-born), annual income (using quintiles, the lowest quintile were those who reported income of < 35 K vs quintile 2–5 ≥ 35 K), education (college or more vs ≤ high school or equivalent), insured (yes vs no), housing status (own vs rent/other arrangements), employed (yes vs no), current treatment (yes vs no), and cancer type (range of multiple cancer sites).

### Health literacy

Health Literacy was assessed using the three-item Brief Health Literacy Screen (BHLS) [26, 27], which measures individual needs for help with filling out forms (“How confident are you filling out medical forms by yourself?”), reading health-related documents (“How often do you have someone help you read health-related materials?”), and difficulty learning due to a lack of understanding of written medical documents (“How often do you have problems learning about your medical condition because of difficulty understanding written information?”). Response options were on a five-point Likert scale, with options for reading and understanding health documents including “Always,” “Often,” “Sometimes,” Occasionally,” and “Never” and for the need for help to fill forms were, “Extremely,” “Quite a bit,” “Somewhat,” “A little bit,” “Not at all.” The survey question measuring if the participant required help with forms was reversed coded. All items were then summed to create a composite score with higher scores (max = 15), indicating fewer health literacy problems. Following a previous study that assessed health literacy using the BHLS scale by Willens and colleagues, we dichotomized this score, with those who scored ≤ 9 as having limited health literacy and scores > 9 as having adequate health literacy [28] (Supplemental Table 1).

### Socioeconomic barriers

Five SES factors (education, income, insurance, housing, and employment status) were dichotomized to create a composite measure following a previous study [29]. If individuals selected an income “≥ 35 K,” an education level of “college or more,” being insured, owning a home, and being employed, they were coded as having no SES barriers (0). Those who selected either one of the following: an income between “ < 35 k,” an educational level of “ ≤ high school or equivalent,” not being insured, not being employed, and/or having a housing status as rent/another arrangement, they were given an additive score ranging from 1 to 5 [29]. Due to sparse counts in categories of four and five SES barriers, scores were truncated to range from 0 to 3 or more SES barriers (Supplemental Table 2).

### Healthcare delays

Nine questions were used to assess healthcare delays. These questions were obtained from the National Health Interview Survey asking participants if they experienced delays in any healthcare received due to various reasons in the past 12 months [25]. These reasons include transportation, living in a rural area where healthcare providers are too far, nervousness about seeing a healthcare provider, could not get time off work, could not get childcare, cannot leave adult unattended due to being a caretaker, could not afford copays, deductible was too high, and could not afford it or had to pay out of pocket for some or all procedures. Response options were “no,” “yes,” or “don’t know.” A dichotomized measure was created with those who responded “yes” to one or more reasons as having experienced healthcare delays, and those who responded “no” to all reasons as having experienced no delays, those who reported “don’t know” were not counted in this measure.

### Statistical methods

To characterize the study population, descriptive statistics were calculated for all demographic variables and variables of interest (nativity, SES barriers, and health literacy). Listwise deletion method was used to address missing data (15.9%). We conducted a post-hoc sensitivity analysis to determine the direction of the potential bias introduced from our listwise deletion method for a complete case analysis. We used a multiple imputation analysis using chained equations (MICE). This approach allowed us to impute missing values based on observed data and estimate relationships between variables. To satisfy the safe data sharing policy of “All of Us,” groups with less than 20 participants are reported in tables as ≤ 20 or < x% with another category in the same column/row also showing ≥ (%) to ensure that another count value cannot be used to derive the exact count that is less than *n* = 20 in the suppressed group. First, a multivariable logistic regression model tested the hypothesized independent relationships between SES barriers, and health literacy with healthcare delays adjusted for covariates (age, sex, ethnicity/race, marital status, treatment status, and cancer type). Secondly, to assess nativity differences in the association between SES barriers and healthcare delays, a product interaction term for nativity and SES barriers (SES barriers*nativity) was included in the model. Furthermore, we assessed for a p-trend in the adjusted and stratified model by introducing SES barriers and health literacy as continuous measures in the model. All statistical analyses were performed using R Jupyter Notebooks embedded in the “All of Us” workbench, with a significance level at alpha 0.05. Odds ratios (ORs) with 95% confidence intervals (CI) and *p* value are reported.

## Results

The median age of the study population (*n* = 10,020) was approximately 64 (interquartile range [IQR Q1, Q3] 55.5, 71.8) years. The majority of participants were female (66.1%), US-born (92%), and self-identified as White (82.3%). There was a higher distribution of females vs males and other sex, foreign-born vs US-born, and Black cancer survivors vs all other race/ethnicity categories that had three or more SES barriers (Table 1). While a higher proportion of females vs males and other sex, foreign-born vs US-born, and Hispanic cancer survivors vs all other race/ethnicity cancer survivors had one or more healthcare delays (Table 2).

Results from the multivariable-adjusted model showed that neither nativity (OR 1.04, 95% CI [0.87, 1.25]) nor health literacy (OR 1.20, 95% CI [0.89, 1.59]) were statistically significantly associated with healthcare delays (see Table 3). However, when assessing for a p-trend for health literacy, for every one-unit increase in health literacy there was an 8% (OR 0.92, 95% CI 0.89, 0.95) decrease in the likelihood to experience healthcare delays. Furthermore, compared to those who did not have any SES barriers, those who reported two or three or more were 65% (OR 1.65, 95% CI [1.43, 1.90]) and 118% (OR 2.18, 95% CI [1.84, 2.58]) more likely to experience delays in healthcare, respectively.

In addition, using SES barriers as a continuous measure to test for a p-trend, we found that for every one additional barrier increase, there was a 29% increase (OR 1.29, 95% CI [1.23, 1.36]) in the likelihood of experiencing healthcare delays. The association between SES barriers and healthcare delays differs by nativity status (*p*_interaction_ = 0.02). The stratified model by nativity showed that among those who were foreign-born, those who experienced two or three or more SES barriers were almost three times (OR 4.35, 95% CI [2.61, 7.34] vs OR 1.53, 95% CI [1.32, 1.78]) and two times (OR 3.83, 95% CI [2.14, 6.98] vs OR 2.10, 95% CI [1.76, 2.50]) as likely to experience healthcare delays as their US-born counterparts who experienced the same levels of SES barriers, respectively (see Table 3).

Assessing for p-trend in the stratified model by nativity, we found that for every additional SES barrier experienced among foreign-born individuals, they were 72% (OR 1.72, 95% CI [1.43, 2.08] vs OR 1.27, 95% CI [1.21, 1.34]) more likely to experience healthcare delays compared to their US counterparts. Finally, in the stratified model, low health literacy was associated with a 41% (OR 1.41, 95% CI [1.02, 1.97]) increase in the likelihood of healthcare delays, and each one-point increase in health literacy score was associated with a 9% (OR 0.91, 95% CI [0.88, 0.94]) decrease in the odds of healthcare delays among US-born cancer survivors. While for foreign-born cancer survivors, low health literacy was not statistically significantly associated with experiencing healthcare delays (OR 0.66, 95% CI [0.34, 1.25]), and for every one-unit increase in health literacy score there was a 2% (OR 0.98, 95% CI [0.90, 1.07]) decrease in the odds of healthcare delays. Although this association did not reach statistical significance (see Table 3).

## Discussion

Using data from the All of Us research cohort, we aimed to investigate the associations between SES barriers and health literacy with healthcare delays. We also explored whether there were any differences in these associations by nativity. We found that among all cancer survivors, health literacy (binary) and nativity were not statistically significantly associated with healthcare delays. We also found that experiencing 2 or 3+ SES barriers was significantly associated with an increased likelihood of healthcare delays. Further, at equal levels of SES barriers, foreign-born individuals had significantly higher odds of healthcare delays when compared to US-born individuals. Lastly, in our separate models by nativity status assessing for a trend, we found that health literacy was inversely associated with healthcare delays among US-born cancer survivors only.

### Socioeconomic barriers

Our multivariable model suggested that cancer survivors who experienced more than one SES barrier experienced an increase in the likelihood of healthcare delays compared to cancer survivors who experienced no SES barriers. These findings are consistent with previous research that found that low SES cancer survivors are more likely to not receive appropriate follow-up care [30] and that those who experience financial, housing, and employment barriers have a greater likelihood of delaying needed care [31–33]. Similarly, in another study, cancer survivors from low SES who reported lower income and education levels were less likely to have follow-up care discussions with their medical providers [30]. This lack of follow-up discussions can potentially contribute to prolonged delays in preventative care. Regarding education, in a previous study by Gonzalez and colleagues, cancer survivors who had a college degree or higher were more likely to have higher access to care but experienced more delays than those with less than or equal to a high school diploma [34]. Perhaps belonging to a higher educational level improves health literacy, enabling cancer survivors to adequately make an informed decision when seeking the appropriate care needed.

Among cancer survivors, those with low SES barriers are more likely to delay medical care, preventive care (dental and vision care), and not fill prescription medications due to cost-related concerns [32, 35, 36]. Furthermore, as cancer survivors are met with the unexpected financial costs of cancer treatment, they may worry about struggling to meet their housing and household bills payments, food insecurities, and retirement [37], thus, potentially forgoing or delaying the crucial care they are required to enhance their survival. Similarly, housing insecurities are linked with negative health outcomes and poor access and quality of healthcare [31, 38]. Lastly, the impact of modifiable factors such as education and SES are inversely associated with experiencing more unmet healthcare needs [39] and a lack of health insurance [39, 40]. Hence, uninsured cancer survivors have a higher risk for comorbidities, bearing a greater mortality risk than uninsured non-cancer survivors [41].

### Nativity differences

Previous research among cancer survivors has shown nativity to be a factor in cost-related barriers among US-born Hispanic cancer survivors [42]. In our study, we found that foreign-born individuals who experienced the same level of SES barriers had higher likelihood of experiencing healthcare delays than their native-born counterparts when compared to those who experienced no SES barriers. Previous research that explored nativity differences among cancer survivors is limited and inconsistent. For example, although the results did not reach statistical significance, Diamant and colleagues reported the opposite in a sample of non-cancer survivors, showing that non-native-born were less likely to report healthcare delays compared to native-born individuals [43]. Whereas, in a study of female cancer survivors that assessed disparities in healthcare access and utilization, they found that non-US-born females were less likely to report having a routine place to go to meet their healthcare compared to US-born cancer survivors [44]. This is important, as not having a primary healthcare office to seek care or have routine services can promote delaying accessing the extended care cancer survivors need. In addition, a higher prevalence of sociodemographic and SES barriers (e.g., income, education) was found among foreign-born individuals than among native-born in two North American countries (US and Canada), and disparities in healthcare access were higher among foreign- compared to native-born [20]. Thus, supporting the role of SES barriers among foreign-born individuals.

### Health literacy

Our study found that, after adjusting for confounding sociodemographic, nativity, and SES barriers, health literacy was not statistically significantly associated with healthcare delays in our entire study cohort. However, we found that solely among US-born participants, limited health literacy was associated with an increased likelihood of healthcare delays compared to adequate health literacy. We also saw a statistically significant monotonic relationship between increased health literacy scores and decreased odds of healthcare delays among US-born. While our findings only reached statistical significance among US-born cancer survivors, the magnitude of our findings was in the same direction among foreign-born cancer survivors. This indicates that regardless of nativity, increasing health literacy could mitigate the impact of healthcare delays. While we controlled for nativity, sociodemographic, and SES barriers, our findings suggest that health literacy could also be associated with cultural differences and language that we were not able to control in our study. For example, cancer survivors with low health literacy may suffer difficulties when trying to decode their symptoms or understand their diagnoses through communication with providers, which could result in healthcare delays and a later stage of disease at diagnosis [45]. In the case of cancer survivors, there is a great need for complex health services after they have completed their primary treatment for their illness and subsequent management of their health [46]. These deficiencies in communicating efficiently with their providers could increase the risk of healthcare delays.

### Strengths and limitations

An important strength of this study is using data from the All of Us research program, as it has a high proportion of enrolled underrepresented minority populations, which increases our sample size and access to geographically and ethnically diverse populations. Similarly, we were able to analyze a relatively large sample with complete socioeconomic and sociodemographic data of foreign-born cancer survivors, which helps to expand the current cancer survivor, cancer health disparities, and health disparities research. Lastly, our results can be generalized to cancer survivors and individuals with similar characteristics and settings that experienced similar SES barriers in the US.

This study is not without limitations. The cross-sectional nature of the design does not allow us to establish a temporal or causal relationship. There is potential for misclassification for some of the factors included in our main independent variables as this data is from self-reported questionnaires. Our sample was also comprised of a higher distribution of individuals with a college degree or higher and a higher income. We were unable able to assess for acculturation; thus, future studies should account for it as it can be a potential confounder in these associations. Using a complete case (CC) analysis method may have introduced bias to our results. We addressed this concern by conducting a sensitivity analysis using multiple imputation (MI) method for our outcome variable. This analysis revealed that there was a slight overestimation in the relationship between SES barriers and healthcare delays among foreign-born cancer survivors, compared to their US-born counterparts with the same level of SES barriers. Despite the small differences observed in the effect estimates, the overall trend and interpretation of the results remained consistent across both the MI and CC analyses (Supplemental Table 3).

Although the All of Us collects data nationwide, these results cannot be generalizable to all cancer survivors in the United States. A clear example of this limitation can be seen in that our sample reported roughly 90% some college or higher degree, whereas in 2021, those who had completed some college, or more were approximately 63% in the general US population [47]. Lastly, an additional limitation is that during our study period, the COVID-19 pandemic may have worsened SES barriers and healthcare delays that may have contributed to our findings and may need further exploring.

### Implications and future direction

Our results can help guide policymakers to promote the development of policies that aim at eliminating SES barriers. For example, many of the variables that are part of our SES index are system-modifiable factors. Implementation of laws that make education equitable, job creation and training, housing affordability, and universal healthcare are ways in which policies can aid in mitigating these SES barriers. At the healthcare system level, practitioners and systems should recognize that these SES barriers exist and promote solutions. For example, systems can offer transportation services to those who are experiencing SES barriers to lessen healthcare delays [48]. Similarly, providing adult- and childcare services can help avoid delays in seeking care [49].

As the All of Us continues to enroll participants and participants complete all surveys, future studies should reassess this association to determine if our findings remain true. Moreover, in future analyses, it is important to consider adjusting for acculturation, as well as other types of stressors such as discrimination, as these experiences can contribute to healthcare delays. Additionally, future studies should aim to explore racial differences between US-born and foreign-born cancer survivors. It is crucial to recognize that races and ethnicities such as Black, Hispanic, and Asian are heterogeneous, varying across cultural and socioeconomic aspects. Thus, understanding the SES barriers associated with healthcare delays among US-born ethnic minorities from different racial and ethnic backgrounds (e.g., Mexican-US-born, Guatemalan-US-born, Chinese-US-born, Nigerian-US-born) compared to foreign-born counterparts (e.g., Mexican-foreign-born, Guatemalan-foreign-born, Chinese-foreign-born, Nigerian-foreign-born) is of extreme importance.

## Conclusion

Using data from the All of Us research program, we found that SES-related barriers are significantly associated with healthcare delays in cancer survivors in our study. However, a greater impact was observed among those who were foreign-born. Similarly, we observed a possible protective effect of health literacy on healthcare delays among US-born only. Our study highlights that to mitigate the impact of delayed healthcare, both policymakers and healthcare providers must prioritize addressing the social determinants of health and promoting health literacy in these populations.

## Supplementary Material

2

## Figures and Tables

**Table 1:** Descriptive characteristics of the sample and their association with SES barriers (*n* = 10,020)

Variables	Number of socioeconomic barriers *n* (%)				*p* value
	No barriers (*n* = 3,112)	1 (*n* = 4,314)	2 (*n* = 1,514)	3 or more (*n* = 1,080)	
Sex					
Female	> 2100 (> 30%)	2688 (40.6%)	> 1000 (> 15%)	> 500 (> 10%)	
Male	991 (29.7%)	1596 (47.9%)	450 (13.5%)	295 (8.9%)	< 0.001
Other^a^	≤ 20 (< 25%)	31 (47.7%)	≤ 20 (< 20%)	≤ 20 (< 20%)	
Age					
Median [Q1, Q3]	59.3 [51.3, 65.8]	68.1 [60.6, 73.2]	64.7 [52.9, 72.0]	58.9 [48.0, 67.3]	< 0.001
Race/ethnicity					
Asian	70 (35.7%)	> 50 (> 40%)	> 20 (> 10%)	≤ 20 (< 20%)	
Black	113 (17.6%)	178 (27.7%)	150 (23.4%)	201 (31.3%)	
Hispanic	143 (23.4%)	164 (26.7%)	137 (22.3%)	171 (27.8%)	
White	2687 (32.6%)	3759 (45.6%)	1154 (14.0%)	643 (7.8%)	< 0.001
More than one pop	52 (34.7%)	> 50 (> 40%)	≤ 20 (< 20%)	≤ 20 (< 20%)	
Other^b^	47 (26.9%)	62 (35.4%)	32 (18.3%)	34 (19.4%)	
Annual income^e^					
< 35 K	≤ 20 (< 20%)	140 (7.7%)	703 (38.4%)	> 900 (> 40%)	
≥ 35 K	2864 (39.6%)	3687 (51.0%)	641 (8.9%)	39 (0.5%)	< 0.001
Other^c^	> 200 (> 20%)	488 (50.8%)	170 (17.7%)	≤ 60 (< 10%)	
Marital status					
Single	> 600 (> 15%)	1215 (35.1%)	778 (22.5%)	> 700 (> 20%)	
Married/living with a partner	2405 (37.2%)	3071 (47.5%)	709 (11.0%)	282 (4.4%)	< 0.001
Other^c^	≤ 20 (< 30%)	29 (30.9%)	27 (28.7%)	≤ 20 (< 10.0%)	
Employed					
Yes	> 2700 (> 50%)	> 900 (> 20%)	> 250 (> 5%)	< 100 (< 5%)	
No	≤ 20 (< 20%)	3273 (60.4%)	1161 (21.4%)	981 (18.1%)	< 0.001
Other^c^	48 (55.2%)	≤ 20 (< 20%)	≤ 20 (< 20%)	≤ 20 (< 20%)	
Educational level					
College or more	3097 (34.5%)	4133 (46.0%)	> 1000 (> 10%)	> 500 (> 5%)	
≤ High school or equivalent	≤ 20 (< 20%)	160 (16.2%)	> 300 (> 30%)	484 (49.1%)	< 0.001
Other^c^	≤ 20 (< 30%)	22 (40.0%)	≤ 20 (< 20%)	≤ 20 (< 20%)	
Insured					
No	≤ 20 (< 30%)	≤ 20 (< 30%)	29 (22.1%)	> 50 (> 50%)	
Yes	> 2500 (> 30%)	> 4000 (> 40%)	1463 (14.9%)	975 (9.9%)	< 0.001
Other^d^	20 (25.3%)	> 20 (30%)	22 (27.8%)	≤ 20 (< 30%)	
Treatment					
No	> 2300 (> 25%)	> 3300 (> 40%)	> 1100 (> 10%)	> 800 (> 10%)	< 0.01
Yes	796 (34.1%)	953 (40.8%)	356 (15.2%)	233 (10.0%)	
Missing	≤ 20 (< 30%)	≤ 20 (< 50%)	≤ 20 (< 25%)	≤ 20 (< 10%)	
Nativity					
US-Born	2869 (31.2%)	4003 (43.5%)	1379 (15.0%)	955 (10.4%)	< 0.001
Foreign-Born	243 (29.8%)	312 (38.3%)	135 (16.6%)	125 (15.3%)	
Housing status					
Own	> 3000 (> 40%)	3534 (47.6%)	< 800 (< 10%)	125 (1.7%)	
Rent/other arrangements	≤ 20 (< 30%)	734 (29.6%)	793 (32.0%)	> 800 (> 35%)	< 0.001
Other^c^	50 (40.0%)	47 (37.6%)	> 20 (> 17%)	≤ 20 (< 30%)	
Cancer type					
Bladder	65 (26.5%)	105 (42.9%)	53 (21.6%)	22 (9.0%)	< 0.001
Blood	238 (31.3%)	350 (46.1%)	111 (14.6%)	61 (8.0%)	
Bone	> 25 (> 25%)	39 (36.5%)	≤ 20 (< 30%)	≤ 20 (< 30%)	
Brain	35 (27.8%)	49 (38.9%)	22 (17.5%)	20 (15.9%)	
Breast	1031 (34.8%)	1283 (43.3%)	408 (13.8%)	240 (8.1%)	
Cervical	188 (27.3%)	202 (29.4%)	126 (18.3%)	172 (25.0%)	
Colon rectal	131 (30.6%)	168 (39.3%)	73 (17.1%)	56 (13.1%)	
Endocrine	≤ 20 (< 30%)	31 (46.3%)	≤ 20 (< 30%)	≤ 20 (< 30%)	
Endometrial	70 (25.6%)	128 (46.7%)	50 (18.3%)	26 (9.5%)	
Head neck	> 50 (> 30%)	79 (42.9%)	25 (13.6%)	≤ 20 (< 10%)	
Kidney	89 (30.4%)	122 (41.6%)	48 (16.4%)	34 (11.6%)	
Lung	39 (16.1%)	109 (45.0%)	55 (21.7%)	39 (16.1%)	
Other	427 (29.8%)	611 (42.6%)	211 (14.7%)	186 (13.0%)	
Ovarian	60 (27.3%)	89 (41.5%)	29 (13.2%)	42 (19.1%)	
Prostate	382 (28.3%)	727 (53.9%)	165 (12.2%)	74 (5.5%)	
Thyroid	245 (38.2%)	223 (34.7%)	104 (16.2%)	70 (10.9%)	
Healthcare delay					
No	2178 (31.6%)	3286 (47.7%)	914 (13.3%)	514 (7.5%)	< 0.001
Yes	934 (29.9%)	1028 (32.9%)	600 (19.2%)	566 (18.1%)	
Health literacy					
≤ 9	≤ 20 (< 10%)	52 (25.1%)	> 20 (10%)	126 (44.9%)	< 0.001
> 9	3048 (31.8%)	4188 (43.7%)	1435 (14.9%)	922 (9.6%)	

Single = includes those who reported: divorced, widowed, separated, and never married. p-values were obtained from chi-square and Kruskal–Wallis tests. Per “All of Us” data use agreement policy, groups < 20 participants are shown as ≤ 20 (%) with a corresponding > (%) category to prevent deriving counts < 20 from other values. No all percentages equal to 100

Q1 = Quartile 1(25%), Q3 = Quartile 3(75%)

*SES* socioeconomic include (education, income, insurance, housing, and employment status)

Data values included in these categories:

a Other and missing

b None of this, another population, and prefer not to answer

c Prefer not to answer and missing

d Missing, do not know, and prefer not to answer, NA = Missing

e Income reported in US dollar, Cancer type “other” also includes esophageal, eye, pancreatic, and stomach cancers

**Table 2:** Descriptive characteristics of the sample and their association with healthcare delays (*n* = 10,020)

Variables	No healthcare delay (*n* = 6,892)	Any healthcare delay (*n* = 3,128)	*p* value
Sex			
Female	4239 (64.0%)	2384 (36.0%)	
Male	2608 (78.3%)	724 (21.7%)	< 0.001
Other^a^	45 (69.2%)	20 (30.8%)	
Age			
Median [Q1, Q3]	66.4 [579, 72.3]	57.8 [46.7, 65.7]	< 0.001
Race/ethnicity			
Asian	132 (67.4%)	64 (32.7%)	
Black	374 (58.3%)	267 (41.7%)	
Hispanic	345 (56.1%)	270 (43.9%)	
White	5856 (71.0%)	2387 (29.0%)	< 0.001
More than one pop	89 (59.3%)	61 (40.7%)	
Other^b^	96 (54.9%)	79 (45.1%)	
Annual income^e^			
< 35 K	914 (50.0%)	916 (50.0%)	
≥ 35 K	5300 (73.3%)	1930 (26.7%)	< 0.001
Other^c^	678 (70.6%)	282 (29.4%)	
Marital status			
Single	2103 (60.8%)	1357 (39.2%)	
Married/living with a partner	4734 (73.2%)	1732 (26.8%)	< 0.001
Other^c^	55 (58.5%)	39 (41.5%)	
Employed			
Yes	2911 (64.4%)	1608 (35.6%)	
No	3923 (72.5%)	1491 (27.6%)	< 0.001
Other^c^	58 (66.7%)	29 (33.3%)	
Educational level			
College or more	6273 (69.9%)	2707 (30.1%)	
≤ High school or equivalent	585 (59.3%)	401 (40.7%)	< 0.001
Other^c^	34 (61.8%)	21 (38.2%)	
Insured			
No	50 (38.2%)	81 (61.8%)	
Yes	6798 (69.3%)	3012 (30.7%)	< 0.001
Other^d^	44 (55.7%)	35 (44.3%)	
Nativity			
US-Born	6364 (69.1%)	2841 (30.9%)	0.01
Foreign-Born	528 (64.8%)	287 (35.2%)	
Housing status			
Own	5521 (74.4%)	1898 (25.6%)	
Rent/other arrangement	1296 (52.3%)	1180 (47.7%)	< 0.001
Other^c^	75 (60.0%)	50 (40.0%)	
Treatment			
No	> 5200 (> 65%)	> 2300 (> 30%)	0.96
Yes	1610 (68.9%)	728 (31.1%)	
Missing	≤ 13 (< 65%)	≤ 20 (< 40%)	
Cancer type			
Bladder	193 (78.8%)	52 (21.2%)	< 0.001
Blood	549 (72.2%)	210 (27.8%)	
Bone	67 (62.6%)	40 (37.4%)	
Brain	72 (57.1%)	54 (42.9%)	
Breast	2076 (70.1%)	886 (42.9%)	
Cervical	316 (45.9%)	372 (54.1%)	
Colon rectal	295 (68.9%)	133 (31.1%)	
Endocrine	46 (68.7%)	21 (31.3%)	
Endometrial	172 (62.8%)	102 (37.2%)	
Head neck	129 (70.1%)	55 (29.9%)	
Kidney	198 (67.6%)	95 (32.4%)	
Lung	176 (72.7%)	66 (27.3%)	
Other	979 (68.2%)	456 (31.8%)	
Ovarian	134 (60.9%)	86 (39.1%)	
Prostate	1120 (83.1%)	228 (16.9%)	
Thyroid	370 (57.6%)	272 (42.4%)	
Health literacy			
≤ 9	178 (55.1%)	145 (44.9%)	< 0.001
> 9	6714 (69.2%)	2983 (30.8%)	
SES barrier			
0	2178 (70.0%)	934 (30.0%)	
1	3286 (76.2%)	1028 (23.9%)	
2	914 (60.4%)	600 (39.6%)	
3+	514 (47.6%)	566 (52.4%)	

Single = includes those who reported: divorced, widowed, separated, and never married. *p* values were obtained from chi-square and Mann–Whitney tests. Any healthcare delay includes delays due to transportation, living in a rural area where healthcare providers are too far, nervousness about seeing a healthcare provider, could not get time off work, could not get childcare, cannot leave adult unattended due to being a caretaker, could not afford copays, deductible was too high or could not afford it or had to pay out of pocket for some or all procedures. Per “All of Us” data use agreement policy, groups < 20 participants are shown as ≤ 20 (%) with a corresponding > (%) category to prevent deriving counts < 20 from other values. No all percentages equal to 100

Q1 = Quartile 1(25%), Q3 = Quartile 3(75%)

*SES* socioeconomic

Data values included in these categories:

a Other and missing

b None of this, another population, and prefer not to answer

c Prefer not to answer and missing

d Missing, do not know, and prefer not to answer, NA = Missing

e Income reported in US dollar, Cancer type “other”: includes esophageal, eye, pancreatic, and stomach cancers

**Table 3:** Results from the multivariable regression analysis of risk factors for health care delay and by nativity status among cancer survivors from the All of Us Research Program

Variables	Adjusted odds ratios *n* = 9,820 OR (95%CI)	US-Born *n* = 9,022 OR (95%CI)	Foreign-Born *n* = 798 OR (95%CI)
Nativity			
US-Born	Ref	–	–
Foreign-Born	1.04 (0.87–1.25)	–	–
Health literacy			
p-trend	0.92 (0.89–0.95)***	0.91(0.88–0.94)***	0.98(0.90–1.07)
≤ 9	1.20 (0.89–1.59)	1.41 (1.02–1.97)*	0.66 (0.34–1.25)
> 9	Ref	Ref	Ref
SES barrier factors index			
p-trend	1.29 (1.23–1.36)***	1.27 (1.21–1.34)***	1.72 (1.43–2.08)***
0	Ref	Ref	Ref
1	0.98 (0.88–1.10)	0.97 (0.86–1.09)	1.15 (0.76–1.74)
2	1.65 (1.43–1.90)***	1.53 (1.32–1.78)***	4.35 (2.61–7.34)***
3+	2.18 (1.84–2.58)***	2.10 (1.76–2.50)***	3.83 (2.14–6.98)***

p-trends were obtained by assessing SES barriers and Health Literacy as continuous measures Adjusted odds ratios (OR) for: sex, race/ethnicity, age, marital status, active treatment, and cancer type *SES* socioeconomic, *Ref* reference group, *CI* confidence interval

Significant *p* values ***< 0.001, **< 0.01, *< 0.05

## Data Availability

Data from the All of Us Research Program can only be accessed through the Researcher Workbench (https://workbench.researchallofus.org/login) as per the informed consent of program participants. The investigators are prohibited to share raw-level data of participants in accordance with the user agreement established by this program. Therefore, it is not possible to provide a de-identified dataset for this manuscript.
