# Supplementary material for: Nativity differences in socioeconomic barriers and healthcare delays among cancer survivors in the All of Us cohort
Source: Cancer Causes Control. Author manuscript; Available in PMC 2024 Feb 5. (PMC10787892; doi:10.1007/s10552-023-01782-z)
Supplement: 2 [file NIHMS1930068-supplement-2.pdf]

Supplemental Table 1: Definitions and Classifications of Health Literacy

Variable	Health Literacy
**Item 1: Filling out forms**	Measures individual confidence in filling out medical forms independently.
Response options	5-point Likert Scale of Extremely to Not at all
**Item 2: Reading health-related documents**	Assesses the frequency of receiving assistance with reading health-related materials.
Response options	5-point Likert Scale of Always to Never
**Item 3: Difficulty learning**	Measures the extent to which difficulty in understanding written medical information affects learning about one's medical condition.
Response options	5-point Likert Scale of Always to Never
**Health Literacy Composite**	
Composite score of the sum of items 1-3	Range 3-15
Dichotomous Composite Score	
<9	Low health literacy
≤9	Adequate health literacy

Supplemental Table 2: Definitions and Classifications of SES barriers

SES barrier	Description
**Income**	Dichotomized into "college or more" (coded as 0) and "≤ high school or equivalent" (coded as 1)
**Education Status**	Dichotomized into "≥35K" (coded as 0) and "<35K" (coded as 1)
**Insurance Status**	Dichotomized into "insured" (coded as 0) and "not insured" (coded as 1)
**Housing status**	Dichotomized into "owning a home" (coded as 0) and "rent/another arrangement" (coded as 1)
**Employment status**	Dichotomized into "employed" (coded as 0) and "not employed" (coded as 1)
**SES Composite Score: Sum of income, education, insurance, housing and employment status**	
0	No SES barriers present
1	One SES barrier present
2	Two SES barriers present
3 or more	Three or more SES barriers present

Supplemental Table 3: Pooled results from the sensitivity multivariable regression analysis of risk factors for healthcare delay and by nativity status among cancer survivors from the All of Us Research Program using multiple imputation chained equations (MICE)

Variables	Adjusted Odds Ratios n=11658 OR (95%CI)	US-Born n=10701 OR (95%CI)	Foreign-Born n=957 OR (95%CI)
**Nativity**			
US Born	Ref	-	-
Foreign Born	1.05 (0.88 – 1.25)	-	-
**Health Literacy**			
**p-trend**	0.92 (0.89 – 0.95)***	0.91(0.88 – 0.94)***	0.96(0.88 – 1.05)
≤9	1.17 (0.89 – 1.54)	1.33 (0.98 – 1.80)	0.73 (0.38 – 1.40)
>9	Ref	Ref	Ref
**SES Barrier Factors Index**			
**p-trend**	1.30 (1.24 – 1.37)***	1.27 (1.21– 1.34)***	1.68 (1.39 – 2.02)***
0	Ref	Ref	Ref
1	0.98 (0.87 – 1.11)	0.97 (0.87 - 1.09)	1.14 (0.71 – 1.85)
2	1.64 (1.43 – 1.89)***	1.54 (1.33 – 1.77)***	3.66 (2.26 – 5.94)***
3+	2.17 (1.84 – 2.56)***	2.08 (1.75 – 2.47)***	3.63 (2.03 – 6.49)***

Adjusted odds ratios (OR) for: sex, race/ethnicity, age, marital status, active treatment, and cancer type.
SES = Socioeconomic, Ref= Reference group, CI = confidence interval,
p-trends were obtained by assessing SES barriers and Health Literacy as continuous measures.
Significant P-values *** <0.001, **<0.01, *<0.05
